# Virus and host-associated variations in the interaction of low-pathogenic avian influenza viruses with the epithelial target tissue of the chicken reproductive tract

**DOI:** 10.1186/s13567-026-01799-7

**Published:** 2026-06-25

**Authors:** Basel A. Abukhadra, Frederik Bexter, Sana I. Mohamed, Lonneke Vervelde, Kate Sutton, Sjaak de Wit, Silke Rautenschlein

**Affiliations:** 1 Clinic for Poultry, University of Veterinary Medicine, Hannover, Germany; 2 Department of Virology, Faculty of Veterinary Medicine, Mansoura University, Mansoura, Egypt; 3 Division of Immunology, The Roslin Institute & Royal (Dick) School of Veterinary Studies, University of Edinburgh, Edinburgh, UK; 4 Royal GD, Deventer, The Netherlands; 5 Faculty of Veterinary Medicine, Utrecht University, Utrecht, The Netherlands

**Keywords:** Low-pathogenic avian influenza virus, chicken genotype, innate immunity, importin, chemerin, interferon, inducible nitric oxide synthase

## Abstract

Low-pathogenic avian influenza viruses (LPAIVs) show pathogenesis variations, suggested of being impacted by virus and host characteristics. This study aimed to understand the interaction of virus and host factors in LPAIV reproductive tract infection. Oviduct organ cultures (OOCs) were prepared from two chicken lines, brown layers (BL) and white layers (WL), to compare virus replication, microscopical lesions, and host-immune responses between two atypical virulent LPAIV strains H3N1 (A/chicken/Belgium/460/2019) and H6N1 (A/chicken/Netherlands/917/2010), and an avirulent H9N2 strain (A/chicken/Saudi Arabia/2525/2000). mRNA levels of interferon λ (IFN λ), inducible nitric oxide synthase (iNOS), host factors such as importin (Imp) α3, α8, and chemerin were measured by quantitative reverse transcription polymerase chain reaction (qRT–PCR). High-throughput transcriptomic profiling of innate immune genes using a quantitative polymerase chain reaction (qPCR) array focused on H3N1- and H9N2-infected BL-OOCs. H3N1- and H6N1-infected BL-OOCs showed significantly higher viral loads compared with H9N2-inoculated OOCs (*p* < 0.05). This was associated with higher mRNA levels of innate-immunity related genes, including Pentraxin (*PTX3*) at 48 hours post-infection (hpi) after H9N2 inoculation. Compared with H9N2, H3N1 triggered a stronger yet delayed innate immune response at 48 hpi with an 8- and 256-fold increase in IFN λ and C–C motif chemokine ligand 4 (*CCL4*) expression, respectively, along with upregulation of Imp α3 in BL-OOCs. WL–OOCs were less susceptible to H6N1 with lower viral loads compared with BL-OOCs, which coincided with lower IFN λ expression levels after virus infection (*p* < 0.05). Overall, the OOCs model was a suitable model to demonstrate variable pathogenicity of LPAIV strains across chicken genotypes. Variations in host responses were detected impacting virus replication in a strain-dependent manner.

**Supplementary information:**

The online version contains supplementary material available at 10.1186/s13567-026-01799-7.

## Introduction

Avian influenza viruses (AIVs) pose a significant global challenge to poultry and constitute a risk to human health owing to their zoonotic potential [1]. Based on virulence, low pathogenic AIVs (LPAIVs) and highly pathogenic AIVs (HPAIVs) can be differentiated [2]. LPAIVs are typically associated with subclinical infection or mild clinical symptoms, depending on the bird species [3]. In domestic poultry, the subtype H9N2 is widely distributed, which predominantly exhibits low virulence in chickens under field and experimental conditions [3]. However, some LPAIV strains can cause an atypical pathogenesis, especially in layers, such as the LPAIV H3N1 strain (A/chicken/Belgium/460/2019), which induced a high mortality rate of up to 60%, and 100% drop in egg production in adult laying hens under field and experimental conditions [4, 5]. In contrast, young male layer-type chickens showed no clinical symptoms 7 days post-inoculation with this strain, with low or no viral shedding and low viral loads in the respiratory and intestinal tracts [5]. Young broilers were found to exhibit very mild clinical signs under field conditions after infection with the Belgian H3N1 [4, 5]. In the Netherlands, a LPAIV H6N1 strain (A/chicken/Netherlands/917/2010), with unusually high virulence, was reported. This strain induced 3.2% mortality and a 74% decrease in egg production in commercial layers [6]. The atypical pathogenic phenotype of these H3N1 and H6N1 strains raises questions about how their genetic constitution is driving this shift in virulence.

In vivo infection of chickens with a H9N2 strain revealed lower virus titers in cloacal swabs of brown layers compared with white layer hens [7]. Genotypic differences were observed in other viral infections as well, with inbred broilers displaying higher expression of interferon type I and lower susceptibility to infectious bursal disease virus (IBDV) than the investigated layer chicken line [8]. This suggests genotype-dependent variation, which may be attributed to the immune response. Therefore, the influence of the chicken genotype on virus replication and immune response remains an important research area to better understand virus–host interactions and improve disease control strategies in poultry.

Although LPAIVs mainly replicate in the respiratory and gastrointestinal tract, some LPAIVs were also shown to infect the chicken oviduct, which is associated with a drop in egg production [5, 9]. Sialic acid α2.3-linked galactose (SA α2.3-gal) receptors, which serve as binding sites for AIVs, are highly expressed in the columnar epithelium of the chicken oviduct [10]. In vivo, chickens experimentally infected with a H9N2 (A/Chicken/shaanxi/01/2011), produced high virus titers in the magnum and uterus segments [11]. Immunohistochemistry (IHC) revealed a higher number of LPAIV H3N1-positive cells in the magnum and uterus of experimentally infected adult laying hens, whereas no staining was detected in other organs, such as the trachea and duodenum [5]. Based on these findings, the magnum appears to be one of the most susceptible target structures of the oviduct for LPAIV infection [5, 11]. Chicken oviduct organ cultures (OOCs) prepared from the magnum were shown to be a suitable in vitro model to study virus–host interactions in the reproductive epithelium for several viruses, including AIV, infectious bronchitis virus (IBV), Newcastle disease virus (NDV), and avian Metapneumovirus (AMPV) [12–14].

In chickens, three types of IFNs have been identified: type I (IFN α, IFN β, and newly characterized IFN κ), type II (IFN γ), and type III (IFN λ) [15]. IFN λ plays a key role in mucosal defense against different viruses such as AIV, NDV, and AMPV [14, 16]. Additionally, activated macrophages produce inducible nitric oxide synthase (iNOS), which stimulates nitric oxide production and plays a crucial role in controlling LPAIV as shown during *in ovo* infection with H4N6 [17]. Notably, both parameters (IFN λ and iNOS) are necessary not only for early antiviral defense but also for shaping adaptive immunity through T-cell modulation [17, 18]. Along with the host-immune system, importin (Imp) isoforms have a role in AIV infection and host adaptation. Imp is a host cellular protein with different isoforms, such as Imp α1, α3, α4, and α7 identified in avian and human cells [19, 20]. Imp α1 is involved in the nuclear localization of AIV (H7N7) proteins such as polymerase basic 2 (PB2) and nucleoprotein (NP), promoting viral replication [21]. In mammals, Imp α8 is essential in reproductive processes, with knockout (KO) models showing impaired embryogenesis in cattle and reduced fertility in mice [22, 23]. Little is known about the distribution and regulation of Imp α isoforms in chickens. In poultry, Imp α3 has the highest mRNA expression in turkey oviduct organ cultures compared with the tracheal organ cultures in response to NDV infection [14]. Additionally, chemerin is a protein implicated in chicken reproductive functions, expressed in epithelial cells of the chicken magnum, and influences embryogenesis [24]. Therefore, it is intriguing to investigate how Imp α3, Imp α8, and chemerin are differentially expressed during AIV infection in the reproductive tract.

The goal of this study was to identify factors impacting LPAIV pathogenesis in the context of two different host genotypes of chickens. Chicken OOC cultures derived from two different layer lines (BL versus WL) were infected with LPAIV strains; H3N1 (A/chicken/Belgium/460/2019), H6N1 (A/chicken/Netherlands/917/2010), and H9N2 (A/chicken/Saudi Arabia/2525/2000) to investigate the influence of host-associated immune responses, and reproductive tract-related parameters, on AIV infection.

## Materials and methods

### Viruses and animals

Three LPAIV strains were used: H3N1 (A/chicken/Belgium/460/2019), H9N2 (A/chicken/Saudi Arabia/2525/2000), and H6N1 (A/chicken/Netherlands/917/2010), which were kindly provided by Prof. Dr. S. de Wit from Royal GD, Deventer, the Netherlands [5, 6]. These viruses were utilized within the framework of the FluNuance project coordinated through the International Coordination of Research on Infectious Animal Diseases (ICRAD; 2821ERA20D). The viruses were propagated in the allantoic cavity of 10-day-old specific-pathogen-free (SPF) embryonated chicken eggs [25] and titrated by plaque assay as previously described [26]. Brown layer (Lohmann Brown classic, BL) pullets were purchased at the age of 17–19 weeks (Geflügelzucht Zahrte GmbH & CO. KG, Wrestedt, Germany). Embryonated SPF eggs from a white layer (Lohmann Selected Leghorn, WL) genotype (Valo® BioMedia GmbH, Osterholz-Scharmbeck, Germany) were incubated at the Clinic for Poultry until hatching. All chickens were raised up to sexual maturity in the facilities of the Clinic for Poultry, University of Veterinary Medicine, Hannover, under strict biosecurity conditions and provided with water and commercial feed *ad libitum*. All birds used for the study were tested for influenza A virus antibodies at the time of sacrifice using the hemagglutination inhibition (HI) test following standard procedures [25] and a commercial enzyme-linked immunosorbent assay (ELISA; ID Screen® Influenza A Antibody Competition, ID. Vet, Montpellier, France) according to the manufacturer’s instructions.

All birds in this study were humanely sacrificed in accordance with German Animal Welfare regulations (permission number: TiHo-T-2019-12 and TiHo-T-2022-16).

### Experimental design

The study used OOCs prepared from 25 to 30-weeks-old BL and WL chickens as an in vitro model to investigate the differential host responses to the infection with LPAIV strains (H3N1, H9N2, and H6N1). Only OOCs from birds that tested negative for influenza A antibodies by ELISA (ID Screen^®^ Influenza A Antibody Competition, ID Vet, Grabels, France) and hemagglutination inhibition test by standard laboratory procedures (data not shown) were used for the experiments. Five laying hens from each genotype (BL and WL) were humanely sacrificed for each experiment (permit number TiHo-T-2022). The final dataset used for analysis included one BL-OOCs experiment (*n* = 5 BL chicken donors) and two WL-OOCs experiments (each with *n* = 5 WL chicken donors). Two BL-OOCs experiments, which were also conducted in addition, were not included in the analysis because bird donors showed AIV antibodies, which interfered with virus-replication in OOCs (data not shown). The infection dose for each virus was 10^2^ plaque-forming units (PFU)/mL, which was concluded to be a suitable virus dose to describe the infections without immediate destruction of the epithelial layer of the OOCs as determined in preliminary experiments (data not shown). The sampling time points for all experiments were 1, 24, 48, and 72 h post-inoculation (hpi).

### Oviduct (magnum) organ culture preparation and LPAIV infection

The magnum *ex vivo* explant cultures were prepared from the chicken oviduct as previously described [12, 14]. In brief, the magnum was aseptically collected and transferred to prewarmed organ culture medium (OCM) (Dulbecco’s Modified Eagle Medium (DMEM) low glucose (Sigma–Aldrich, Steinheim, Germany) with penicillin/streptomycin (P/S; 100 U/mL, 100 mg/mL, Sigma). The magnum was opened longitudinally, and the explants were carefully prepared by using tissue punches (Mediware, Wesel, Germany) with a 5 mm diameter, with subsequent cutting into 2–3 mm diameter explants with a microtome blade. All OOC explants (OOCs) were distributed individually into 24-well plates and inoculated using 200 µL of organ culture medium with 10^2^ PFU/mL for each virus and virus-free media for the control group. The inoculation was performed on the day of OOCs preparation. After 1 h of incubation, the virus-containing medium and virus-free medium were removed from OOCs with subsequent washing with phosphate-buffered saline (PBS) (1 mL/well). The OOCs were cultured in 1 mL of OCM supplemented with heat-inactivated 5% fetal bovine serum (FBS, Biochrom, Berlin, Germany), 2% chicken serum (Sigma), 2.5 μg/mL Amphotericin B (Biochrom), and 1% of nonessential amino acids (Biochrom). All plates were incubated on a shaker (GFL, Burgwedel, Germany) at 37 °C and 5% CO_2_ [13, 14]. The ciliary activity of magnum explants was monitored via a bright-field microscope and evaluated to ensure tissue viability throughout the experiment. Five OOCs derived from five different birds were collected per time point for each virus-inoculated and virus-free group. The explants were collected in a small volume of incubation medium used during the experiment and immediately stored at −80 °C until analysis, or they were fixed in 4% formalin for histology.

### RNA isolation

The BL- and WL-OOCs were mechanically homogenized in lysis buffer after removal of culture media. Homogenization was performed using a Precellys^®^ 24-tissue homogenizer (Bertin Technologies, Montigny-le-Bretonneux, France) set to 5000 rotations per minute (rpm) for 20 s. RNA was isolated using the innuPREP*®* RNA mini kit (Analytik Jena GmbH, Jena, Germany) according to the manufacturer’s instructions. The RNA quantity and quality were determined by NanoDrop (DeNovix, Wilmington, USA) and samples with a 260/280 ratio of approximately 1.8 or higher were selected for subsequent analysis. All RNA samples were stored at −80 °C until use.

### Quantitative reverse transcription PCR (qRT–PCR)

Investigation of virus replication kinetics was performed by qRT–PCR detecting the AIV matrix (M) gene [27]. qRT–PCR was conducted with the qScript^TM^ XLT one-step qRT–PCR ToughMix Low ROX-polymerase (Quantabio, Beverly, MA, USA) for viral mRNA quantification and IFN λ, iNOS, Imp α3, Imp α8, and chemerin mRNA expression according to the manufacturer’s instructions [14, 20]. All primers and probes used in the study are listed in (Additional file 1) [27–29]. All samples were investigated in duplicates. The QuantStudio 3 Real-Time cycler (Applied Biosystem, Thermo Fisher Scientific) with previously published PCR thermal profiles was used for AIV quantification, [27] and IFN λ, iNOS [14], and Imp α3 [14, 20] mRNA expression. The mRNA expression analysis for chemerin and Imp α8 was conducted under the following thermal profile: 1 cycle at 55 °C for 10 min and 95 °C for 1 min followed by 40 cycles at 95 °C for 10 s and 60 °C for 1 min.

### Histopathological analysis

Virus-free and virus-infected OOCs were fixed with 4% formalin and embedded in paraffin with subsequent cutting into 2-µm thick sections. The sections were stained with hematoxylin and eosin (HE) following standard procedures [9]. Histopathological lesions were microscopically examined to assess in particular loss of cilia, cell detachment, and cell flattening at the epithelium level in addition to damage to underlying tissue [14]. The assessment was based on a scoring system for each lesion as follows: 0 = no lesion as indicated by no loss of cilia, normal epithelium, and preserved underlying histological architecture; 1 = mild lesion characterized by slight or focal loss of cilia, minor epithelial flattening, no epithelial cell detachment, and minimal damage without disruption of underlying tissue structure; 2 = moderate lesion corresponds to more extensive ciliary loss affecting larger areas, moderate epithelial flattening, partial or localized epithelial cell detachment, and moderate disruption of underlying histological tissue architectures; and 3 = severe lesion marked by nearly complete or complete loss of cilia, pronounced epithelial flattening, extensive cell detachment, and severe tissue damage including loss of normal histological appearance or necrosis [9]. Five rings per virus per time point (24 and 48 hpi) for each genotype (BL and WL) were evaluated, and the average score of each lesion was calculated.

### Dynamic 96.96 qPCR array

#### RNA reverse transcription and complimentary DNA (cDNA) preamplification

RNA samples of H3N1 and H9N2 inoculated- and virus-free-BL-OOCs were further selected to investigate innate immune-related differentially expressed genes (DEGs) by qPCR array at 1, 24, and 48 hpi. The RNA samples were analyzed at the Roslin Institute, University of Edinburgh, UK. The RNA samples were treated with DNAase using a DNA-free^TM^ Kit (Applied Biosystems, Foster City, CA, USA) and then reverse-transcribed with random hexamer primer and oligo (dT) using a High-Capacity Reverse Transcription kit (Life Technologies, Paisley, UK) to form cDNA [30]. cDNA was preamplified by adding 2.5 µL of diluted cDNA (1:5) to 5 μL of TaqMan PreAmp Master Mix (Applied Biosystems) and 2.5 μL of a 200 nM mixed stock pool of primer pairs. Preamplification was performed at 95 °C for 10 min followed by 14 cycles of 95 °C for 15 s and 60 °C for 4 min [30]. 16 U/μL exonuclease I (*E. coli*, New England Biolabs) was used to digest unincorporated primers at 37 °C for 30 min and 80 °C for 15 min.

### Microfluidic dynamic qPCR array

The preamplified cDNAs were investigated by qPCR with the microfluidic 96.96 Dynamic array (Fluidigm array) (Standard BioTools UK Ltd., London, UK), which was performed on a BioMark HD instrument (BioMark) as previously described [31]. The microfluidic dynamic qPCR array targets 86 innate immune-related genes in addition to six reference genes using a thermal profile as previously described [31]. All primers used were previously described [31]**.** The RNA quantification at each cycle based on fluorescence emission was analyzed using the real-time PCR Analysis software v 3.1.3 (Fluidigm).

### Transcriptomic analysis of differentially expressed genes

Expression stability of putative reference genes: beta-actin (ACTB), glyceraldehyde-3-phosphate dehydrogenase (GAPDH), beta-glucuronidase (GUSB), ribosomal 28S (r28S), TATA box binding protein (TBP), tubulin alpha chain (TUBAT), and beta-2-microglobulin (B2M) were evaluated by using the NormFinder tool in GenEx7 (MultiD Analyses AB, Gothenburg, Sweden) [30]. The geometric mean of the most stable genes (B2M, ACTB, TUBAT, and TBP) was used to normalize all samples. The target genes are expressed as log_2_ fold change in relation to time-matched virus-free samples calculated by the 2^−ΔΔCT^ method using GenEx7 (MultiD Analyses AB) [30]. Heatmaps were generated for DEGs with a log_2_ fold change > 1 and <  −1. Additionally, volcano plots were created with statistically significant DEGs (*p* < 0.05) with a log_2_ fold change > 2 and <  −2. Both heatmaps and volcano plots were generated using GraphPad Prism 8.02 (GraphPad Software, Inc., San Diego, CA, USA).

### Data and statistical analysis

The virus loads are presented as mean 40-cycle threshold (Ct) ± SD. Ct values higher than 31 approach the assay’s limit of detection, indicating minimal viral RNA levels, suggesting a lack of active viral replication according to previous validation studies in chickens [32]. The mRNA expression of IFN λ and iNOS, Imp α3, α8, and chemerin is expressed as 40-Ct and log_2_ fold change in relation to virus-free controls using the delta Ct method (ΔCt method). All Ct values are normalized with the detection of 60S ribosomal protein L13 (RPL13) [14, 33]. The Shapiro–Wilk test assessed the normality of the data. Statistically significant differences between time points for each group of normally distributed data were analyzed using analysis of variance (ANOVA), followed by the Tukey’s honestly significant differences (HSD) all-pairwise comparison test (*α* = 0.05). Before performing ANOVA, the assumption of homogeneity of variances was evaluated using Levene’s, O’Brien’s, and Brown–Forsythe tests; the results (*p* > 0.05) indicated that the variances were homogeneous across groups, justifying the use of ANOVA. Significant differences were identified between the LPAIV strains, genotypes, and the virus-free control groups at each time point using the two-sample *t*-test (*p* < 0.05). For comparisons involving non-normally distributed data across different groups, the Wilcoxon signed-rank test was used. Two-way ANOVA test was done on mRNA expression of all investigated parameters for detection of significant virus, genotype, and their interaction effects at each time points (24–72 hpi). All statistical tests were performed using Statistix, Version 10.0 (Analytical Software, Tallahassee, FL, USA).

Statistical analysis of DEGs analyzed via qPCR array between the different groups was performed using GenEx7. The statistical significance was assessed by a two-way *t*-test and adjusted for multiple comparisons using post hoc Bonferroni correction as previously described [30]. The DEGs with *p*-value < 0.05, log_2_ fold change > 2 and <  −2 were considered significantly regulated. In WL experiments, data from two independent experiments were merged to increase the number of biological replicates and ensure robust statistical analysis. In addition, owing to occasional sample loss during experimental processing or exclusion of statistical outliers, the number of replicates per strain in WL model ranged from 5 to 10 with the exact number indicated in the figures.

## Results

### Virus strain and chicken genotype effect on LPAIV replication

**Figure 1:**
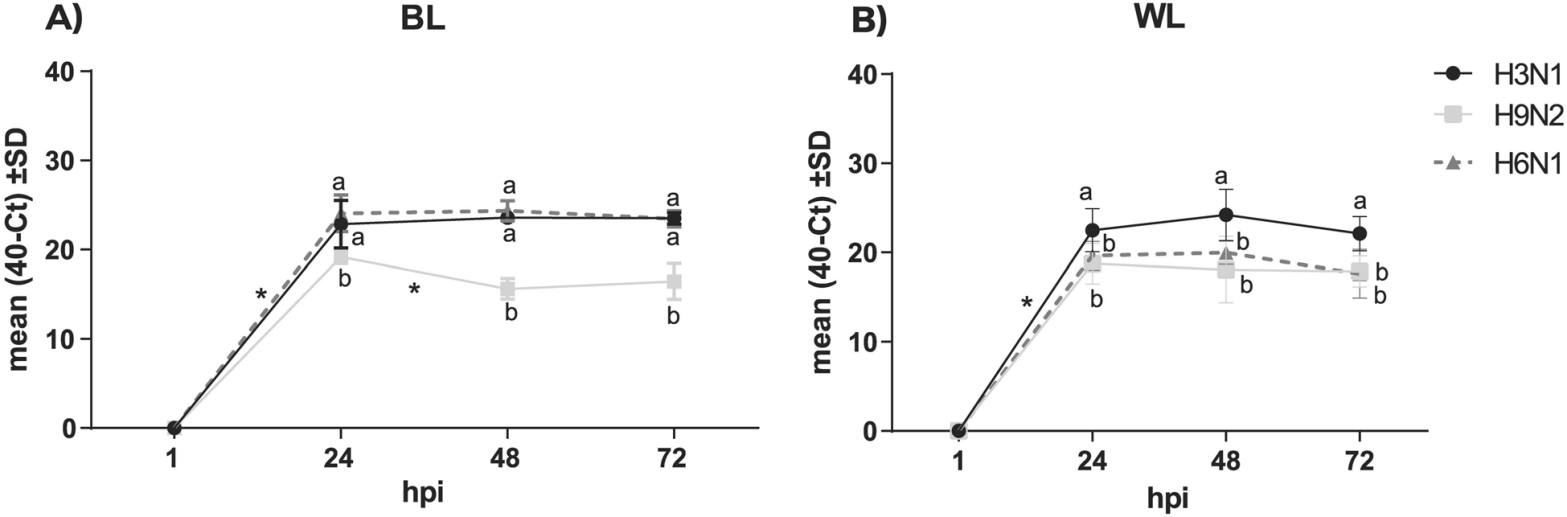
**LPAIVs replication in BL- (A) and WL- (B) OOC**. OOCs were inoculated with either H3N1, N6N1, or H9N2. The viral replication was analyzed by qRT-PCR. Normalized data are presented as mean 40-Ct ± standard deviation (SD). Lowercase letters indicate significant differences between LPAIV strains at the same time point (two-sample *t*-test). Asterisks indicate significant differences for each virus across different time points using ANOVA followed by Tukey HSD all-pairwise comparison test, *p* < 0.05. *n* = 5 OOCs derived from 5 different BL chickens/time point, *n* = 8–10 OOCs derived from 8 to 10 different WL- chickens time/point. hpi: hours post-inoculation.

The viral RNA in OOCs was quantified using qRT–PCR to detect differences in viral replication kinetics between LPAIV strains H3N1, H9N2, and H6N1 and between layer chicken genotypes (BL and WL). Compared with 1 hpi, all investigated strains exhibited significantly higher viral RNA levels at later time points, peaking at 24 and 48 hpi in both genotypes except for H9N2, which peaked at 24 hpi and declined afterwards, particularly in BL-OOCs (*p* < 0.05) (Figure 1A). BL-OOCs showed for both H3N1 and H6N1 strains significantly higher viral loads compared with H9N2 at 24–72 hpi (*p* < 0.05) (Figure 1A). H3N1 demonstrated significantly higher viral replication kinetics across time points (24–72 hpi) compared with H9N2 and H6N1 in WL-OOCs (*p* < 0.05) (Figure 1B). Genotypic differences in H6N1 replication were observed at 24–72 hpi with significantly higher viral RNA levels in the BL-OOCs compared with WL-OOCs (*p* < 0.05) (Additional file 2). Genotype-dependent difference was also observed for H3N1 with significantly higher viral RNA levels in WL-OOCs compared with BL-OOCs at 48 hpi (*p* < 0.05) (Additional file 2). No significant differences were found between genotypes for H9N2 at all investigated time points (Additional file 2). At 24 hpi, the two-way ANOVA analysis revealed a significant effect of virus strains and chicken genotypes (*p* < 0.05), with no significant interaction between them (Additional file 3). At 48 and 72 hpi, the virus replication was significantly influenced by virus strains; however, there was no significant effect of genotype. Instead, a significant interaction between virus strain and genotype was observed (Additional file 3).

### Histopathology

**Figure 2:**
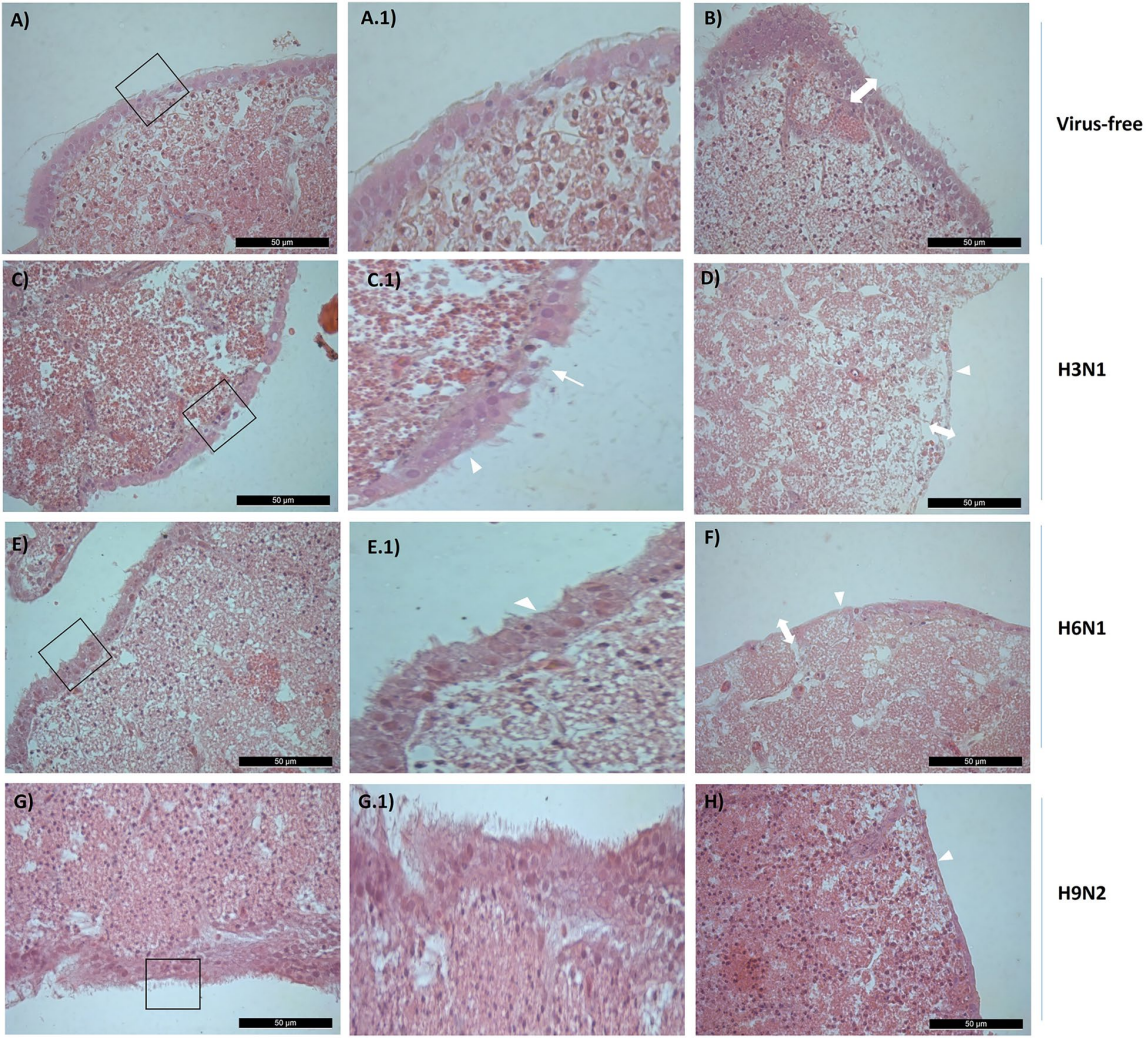
**Histopathological analysis of LPAIV-infected BL-OOCs at 24 and 48 hpi by HE staining. **(A, B) virus-free OOCs, H3N1- (C, D), H6N1- (E, F), and H9N2-infected OOCs (G, H) at 24 and 48 hpi. Black boxes represent the zooming area shown in A.1, C.1, E.1 and G.1 of A, C, E, and G, respectively. Arrows indicate destruction of epithelial cells. Arrow heads point out the loss of cilia. Double-headed arrows point out flattening of the epithelial cells. 400× fold magnification is used, and the scale bar is 50 µm.

We analyzed the effect of LPAIV H3N1, H6N1, and H9N2 strains on BL- and WL-OOCs lesion development at 24 and 48 hpi. In virus-free BL- and WL-OOCs, an intact epithelial cell layer was observed with cilia present at 24 and 48 hpi (Figure 2A and B). In H3N1- and H6N1-infected BL-OOCs, severe loss of cilia was observed at 24 hpi with complete ciliary loss by 48 hpi (Figure 2C–F) (Table 1). Destruction and partial detachment of the epithelial cells are clearly evident in H3N1-infected BL-OOCs at 24 hpi (Figure 2C). H3N1- and H6N1-infected BL-OOCs demonstrated pronounced flattening of epithelial cells compared with H9N2-infected OOCs and virus-free controls at 48 hpi (Figure 2D and F) (Table 1). In addition, H3N1 induced severe disruption of structure and necrosis to the underlying lamina propria of the BL-OOCs by 48 hpi, which was present, but less severe for the H6N1-infected BL-OOCs (Figure 2D and F) (Table 1). For H9N1-infected BL-OOCs, loss of cilia is evident at 48 hpi compared with 24 hpi with little to no damage to the underlying tissue (Figure 2G and H).

**Table 1:** **Histological lesions in OOCs of BL and WL infected with LPAIVs**

OOC-genotype	Virus	Time point	Average lesion score of 5 explants/time point and group			
			Loss of cilia	Epithelial cell detachment	Epithelium flattening	Damage of underlying tissue
BL	H3N1	24 hpi	3	2	2	3
		48 hpi	3	3	3	3
	H6N1	24 hpi	3	0	0	2
		48 hpi	3	0	2	2
	H9N2	24 hpi	1	0	0	1
		48 hpi	3	0	0	1
WL	H3N1	24 hpi	3	3	3	0
		48 hpi	2	0	3	3
	H6N1	24 hpi	1	0	0	0
		48 hpi	2	0	2	2
	H9N2	24 hpi	2	0	0	0
		48 hpi	1	0	1	1

The table summarizes the histopathological lesion development in oviduct organ cultures (OOCs) of two different chicken genotypes, brown layer (BL) and white layer (WL), infected with H3N1, H9N2, and H6N1 strains. Lesions were assessed using a semi-quantitative scoring system of each lesion (0 = no lesion, 1 = mild, 2 = moderate, 3 = severe lesions). Mild lesion characterized by focal loss of cilia, normal epithelium with slight flattening, and no damage to underlying tissue. Moderate lesion characterized by severe loss of cilia, focal epithelial cell detachment, and moderate disruption of underlying tissue. Severe lesion characterized by complete ciliary loss, extensive epithelial cell detachment, and severe disruption of the histological structure of the underlying tissue with extensive necrosis. Underlying tissue refers to the lamina propria. The scores in the table represent the average lesion score across five explants/virus-infected or virus-free/genotype/time point

WL-OOCs showed epithelial cell flattening and clumping of cilia in response to H3N1 infection compared with intact cilia observed for H6N1 and H9N2 at 24 hpi. At 48 hpi, WL-OOCs showed severe epithelial cell flattening in response to all strains except H9N2 compared with virus-free controls (Table 1). Loss of cilia was evident in H3N1- and H6N1-infected WL-OOCs while they were still present after H9N2 infection at 48 hpi (Table 1). In addition, loss of tissue architecture of the lamina propria was evident in H3N1-infected WL-OOCs at 48 hpi (Table 1). Overall, irrespective of genotypes, H3N1 and H6N1 induced severe damage to epithelial cells, while H9N2 showed a mild destructive effect on OOCs (Table 1).

### Role of genetic background in differential mRNA expression of IFN λ and iNOS in LPAIV-infected OOCs

**Figure 3:**
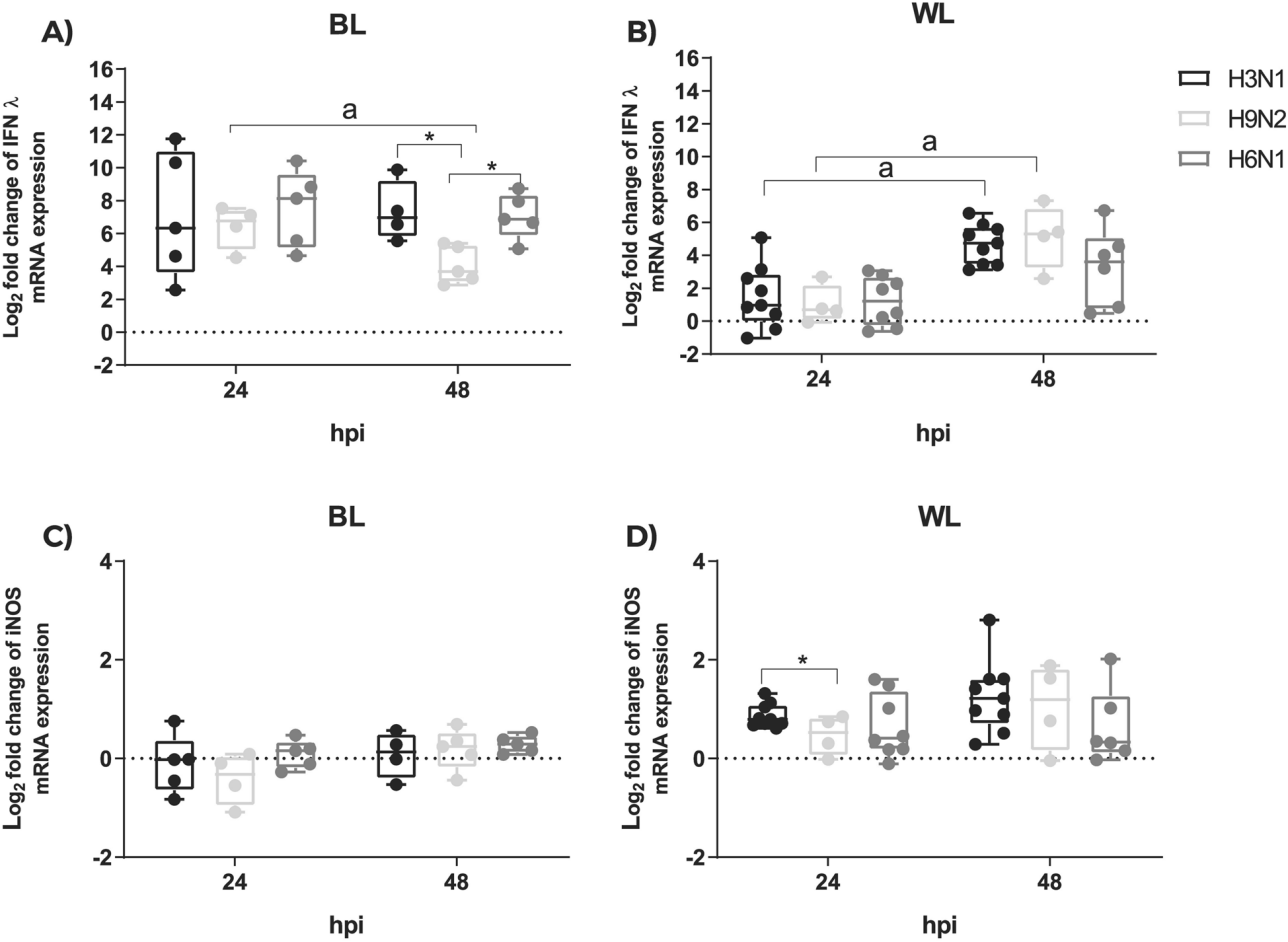
**Influence of genetic background and virus strain on IFN λ and iNOS mRNA expression**. IFN λ (A, B) and iNOS (C, D) mRNA expression levels were investigated in LPAIV-inoculated OOCs derived from BL (A, C) and WL (B, D) chickens. Error bars indicate standard deviations (SD). Asterisks indicate significant differences between virus strains at the same time point (two-sample *t*-test). Lowercase letters indicate significant differences between different time points for each virus-inoculated group (two-sample *t*-test). *p* < 0.05; *n* = 5 OOCs derived from 5 different BL chickens/time point, *n* = 8–10 OOCs derived from 5 to 8 different WL- chickens/time point. hpi: hours post-inoculation.

To assess the mRNA expression levels of IFN λ and iNOS in LPAIV-inoculated BL- and WL-OOCs, qRT–PCR was performed on virus-free and infected OOCs at time points 24 and 48 hpi, which were selected to capture the peak of virus loads, basal IFN λ mRNA expression levels in virus-free samples of BL-OOCs were lower compared with WL-OOCs (Additional file 4A). All infected BL-OOCs exhibited a significant upregulation of IFN λ compared with the virus-free group at 24 and 48 hpi (*p* < 0.05) (Figure 3A) (Additional file 4A). A similar trend was observed in LPAIV-infected WL-OOCs, which was significant at 48 hpi (*p* < 0.05) (Figure 3B) (Additional file 4A). We observed strain variations in IFN λ mRNA expression at 48 hpi in LPAIV-inoculated BL-OOCs with significantly higher expression in H3N1- and H6N1-inoculated OOCs, showing an eight-fold increase compared with H9N2-inoculated OOCs (*p* < 0.05) (Figure 3A). By comparing genotypes, BL-OOCs exhibited significantly higher IFN λ mRNA levels by nearly 45- and 8-fold at 24 and 48 hpi, respectively, compared with WL-OOCs in response to all investigated strains except for H9N2, for which significant differences were only observed at 24 hpi (*p* < 0.05) (Figure 3A and B).

iNOS mRNA expression was compared between LPAIV-inoculated and virus-free OOCs (Figure 3C and D) (Additional file 4B). iNOS mRNA levels were mostly comparable between infected and virus-free groups, particularly in LPAIV-inoculated BL-OOCs at both time points (Figure 3C). However, WL-OOCs exhibited significant upregulation in response to H3N1 at 24 hpi and to H3N1 and H9N2 infection at 48 hpi compared with virus-free controls (*p* < 0. 05) (Additional file 4B). H3N1-inoculated WL-OOCs showed statistically significant upregulation of iNOS mRNA compared with H9N2 at 24 hpi (*p* < 0. 05) (Figure 3D). Statistical analysis by two-way ANOVA test showed a significant impact of chicken genotype on IFN λ and iNOS mRNA expression at 24 and 48 hpi while no direct virus effect was observed. A significant impact was observed for the combination of genotype and virus-strain at 48 hpi for IFN λ mRNA expression (Additional file 3).

### High-throughput innate immune-related gene expression profiling using 96.96 dynamic qPCR array

**Figure 4:**
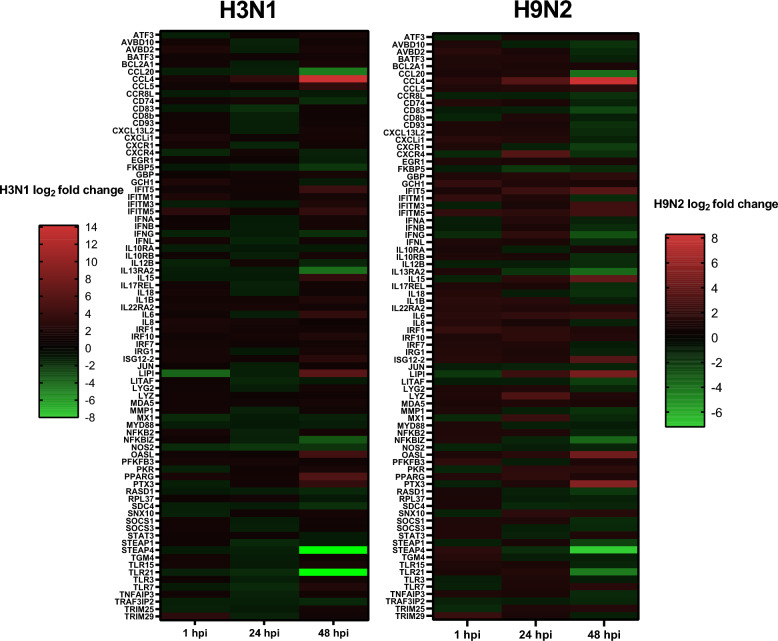
**Heatmap reveals distinct gene expression patterns in response to H3N1 and H9N2 infection of BL-OOCs**. The heatmap shows log_2_ fold changes in gene expression relative to mock-infected. Each column represents DEGs at a single time point, such as 1, 24, and 48 hpi. Upregulated genes are shown in red (log_2_ fold change > 1), and downregulated genes in green (log_2_ fold change < −1). The divergence in color intensities indicates magnitude of log_2_ fold change values of upregulated and downregulated genes. *n* = 5 OOCs derived from 5 BL-chickens/virus/time point.

**Figure 5:**
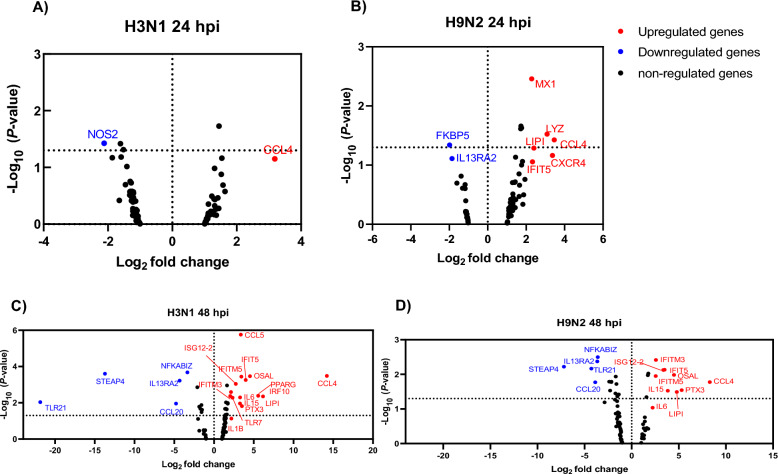
**Volcano plots showing DEGs in LPAIV-inoculated OOC of BL chicken compared with virus-free controls**. DEGs were investigated in H3N1 (A, C) and H9N2 (B, D) inoculated OOC explants of BL chickens (*n* = 5 OOCs derived from five different BL-chickens) at 24 and 48 hpi, respectively, in comparison with time-matched virus-free controls. Each dot represents an individual gene. DEGs are considered significant when log_2_ fold change is > 2 or < −2 with a *p*-value < than 0.05. Upregulated genes are highlighted in red and downregulated genes are shown in blue, while nonregulated genes appear in black.

In our initial analysis of innate immune responses, the BL-OOCs induced higher mRNA levels of the selected genes such as IFN λ compared with WL-OOCs. Therefore, to take a closer analysis of the differences in host immune response, we analyzed the mRNA expression levels of 86 antiviral innate immune genes comparing atypical virulent H3N1 and avirulent H9N2 LPAI strains using 96.96 dynamic array. Compared with virus-free controls, both strains induced a comparable number of upregulated genes at 1 hpi (Additional file 5). The heatmap of the log_2_ fold change levels of time-matched infected versus virus-free OOCs, demonstrated strain-dependent variations in the induction of innate immune gene expression in response to H9N2 compared with H3N1 at 24 hpi (Figure 4). Differential gene expression analysis revealed that at 24 hpi, 56/86 (65%) immune-related genes were upregulated in response to H9N2 infection, while only 31 genes (36%) were upregulated in response to H3N1 infection compared with virus-free controls (Figure 4) (Additional file 5). However, at 48 hpi, H3N1 induced a higher number of upregulated genes 65%, compared with 33% in H9N2 (Additional file 5). Volcano plots were generated to visualize the genes that were significantly regulated by viral infection. H9N2 induced significant upregulation of *CCL4*, *LIPI*, *CXCR4*, *LYZ*, *MX1*, and *IFIT5* compared with virus-free controls at 24 hpi (*p* < 0. 05), which were not detected in H3N1 infection except for *CCL4* (Figure 5A and B). The 48 hpi samples were characterized by the highest magnitude of significantly upregulated and downregulated gene expression levels compared with the earlier time points (Figure 4) (Figure 5C and D). At 48 hpi, both infected groups exhibited significant upregulation of shared immune-related genes, including *CCL4*, *IFIT5*, *IFITM3*, *IFITM5*, *IL15*, *IL6*, *ISG12-2*, *LIPI*, *OASL*, *PPARG*, and *PTX3* as well as downregulation of *CCL20*, *IL13RA2*, *NFKBIZ*, *STEAP4*, and *TLR21* compared with virus-free controls (*p* < 0 0.05) (Figure 5C and D) (Additional file 5). However, the magnitude of regulation compared with virus-free controls varied between the two viruses, particularly at 48 hpi, as H3N1-infected OOCs had higher transcript levels of *CCL4* and lower levels of *TLR21* and *STEAP4* compared with the levels in the H9N2-inoculated group at 48 hpi (Figure 5C) (Additional file 5). Unlike H9N2, H3N1 infection induced a distinct innate immune signature characterized by significant upregulation of *CCL5*, *PPARG*, *IL6*, and *TLR7* at 48 hpi (*p* < 0 0.05). Additionally, compared with virus-free controls, H9N2 exhibited higher levels of *ISG12-2* and *PTX3* mRNA with a 1.5- and 3.5-fold increase, respectively, compared with H3N1 at 48 hpi (*p* < 0 0.05) (Figure 5D) (Additional file 5).

### Importin α3, importin α8, and chemerin cellular response variations against LPAIV infection of BL and WL OOCs

**Figure 6:**
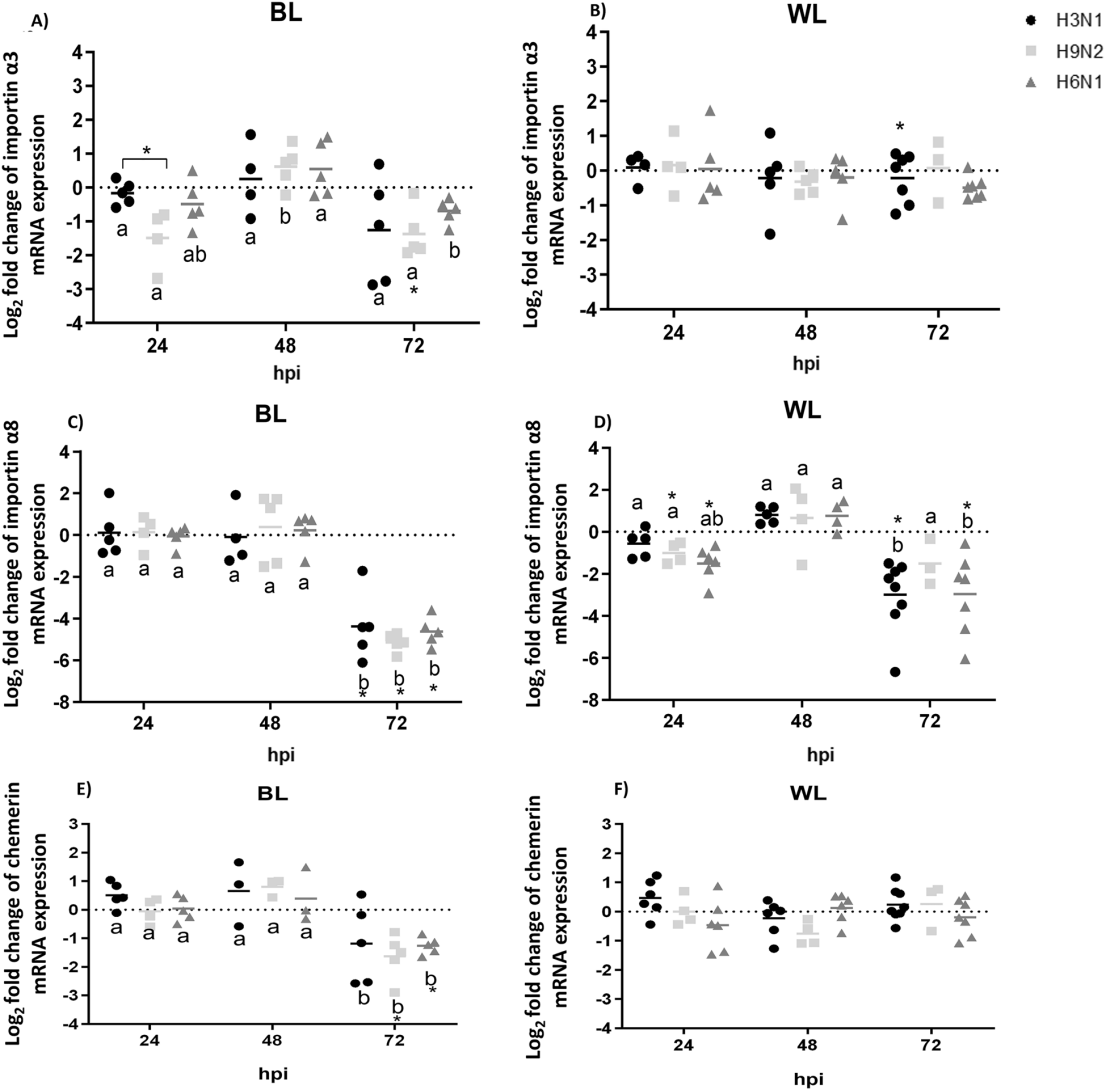
**mRNA quantification of importin α α3, α8, and chemerin using qRT–PCR**. Importin α3 (A, B), importin α8 (C, D), and chemerin (E, F) mRNA expression is shown as log_2_ fold change. The mRNA expression was investigated in response to H3N1, H9N2, and H6N1 infection of OOC explants of BL chickens compared with WL at 24 and 48 hpi. Asterisks indicate significant differences between virus strains or between virus-inoculated and virus-free groups at the same time point. Lowercase letters indicate significant differences between different time points for each virus-inoculated group using ANOVA followed by Tukey HSD all-pairwise comparison test. *p* < 0.05; *n* = 5 OOCs derived from 5 different BL chickens/time point, *n* = 5–8 OOCs derived from 5 to 8 different WL- chickens/time point. hpi: hours post-inoculation.

Owing to their role in viral replication and reproductive functions in chickens and mammals, Imp α3, α8, and chemerin mRNA expression levels were assessed by qRT–PCR in LPAIV-inoculated BL- and WL-OOCs and were compared with virus-free controls. Imp α3 basal expression levels in virus-free OOCs were lower in BL-OOCs compared with WL-OOCs, while no genotype effect was detected at the basal levels of Imp α8 and chemerin (Additional file 6A and B). Downregulation of Imp α3 mRNA expression was observed in H9N2-infected BL-OOCs at 24 and 72 hpi compared with virus-free OOCs (*p* < 0.05) (Figure 6A). H9N2-infected BL-OOCs showed statistically significantly lower Imp α3 mRNA expression levels compared with H3N1 at 24 hpi (*p* < 0.05) (Figure 6A).

H3N1-infection of WL-OOCs induced a significant downregulation of Imp α3 compared with virus-free controls at 72 hpi (*p* < 0.05) (Figure 6B) (Additional file 6A). The chicken genotype influenced the expression of Imp α3 in response to H9N2 in BL-OOCs with a statistically significant downregulation by 3- and 2.5-fold at 24 and 72 hpi, respectively, compared with WL-OOCs (*p* < 0.05) (Figure 6A and B). In addition, significant upregulation of Imp α3 mRNA expression was observed in H9N2-infected BL-OOCs by nearly 2-fold compared with WL at 48 hpi (*p* < 0.05) (Figure 6A and B).

Significant downregulation of Imp α8 expression was observed in BL- and WL-OOCs in response to the H3N1 and H6N1 strains at 72 hpi (*p* < 0.05) (Figure 6C and D). Genotype-dependent variations were observed by significant downregulation of Imp α8 mRNA expression in H9N2- and H6N1-infected WL-OOCs, compared with the virus-free OOCs and with BL-OOCs at 24 hpi (*p* < 0.05) (Figure 6C and D) (Additional file 6B). Additionally, statistically significant downregulation of Imp α8 was evident in H9N2-infected BL-OOCs compared with WL at 72 hpi (*p* < 0.05) (Additional file 6B).

The expression of chemerin mRNA in OOCS showed minor upregulation in some individual replicates in response to H3N1 compared with the other investigated strains at 24 hpi in both BL- and WL-OOCs (Figure 6E and F). BL-OOCs demonstrated significantly lower mRNA expression levels of chemerin in H9N2- and H6N1-infected OOCs compared with virus-free controls at 72 hpi (*p* < 0.05) (Additional file 6C). We observed genotype-specific differences, when BL-OOCs expressed significant upregulation of the chemerin mRNA level by 2.8-fold in response to H9N2 compared with no change after WL-OOCs infection at 48 hpi (*p* < 0.05) (Figure 6E and F) (Additional file 6C) (Figure 6E and F). Two-way ANOVA statistical test showed that a genotype-dependent effect is evident at 24 hpi on Imp α3 and Imp α8 mRNA expression while a virus strain effect was observed only for chemerin mRNA expression at this time point. At 48 hpi, the genotype mainly affected mRNA expression of Imp α3 and chemerin. At 72 hpi the differential mRNA expression of Imp α3, Imp α8, and chemerin is mainly affected by genotype differences. No significant virus genotype interaction was observed for these parameters at all investigated time points (Additional file 3).

## Discussion

Little is known about the interaction of LPAIV with chicken reproductive epithelial cells, although various field and experimental studies have indicated that different LPAIVs target the chicken oviduct with variable magnitudes of production losses [5, 11, 12]. Our study used an OOC in vitro model to investigate possible variations in viral replication and pathogenicity associated with LPAIV strains of different virulence and between two chicken genotypes. In addition, we investigated mRNA expression of Imp α3, α8, and chemerin, as reproductive tract-associated factors in response to LPAIV infection.

Viral RNA loads peaked for all investigated strains in OOCs of both chicken genotypes at 24 hpi, confirming the magnum as a target site for LPAIV infection [5, 12].

Notably, the H3N1 strain showed significantly higher viral loads compared with H6N1 and H9N2 in WL-OOCs, whereas in BL-OOCs, H3N1 and H6N1 showed higher viral RNA levels than H9N2 at 24–72 hpi (*p* < 0.05), suggesting a genotype-specific impact on H6N1 infection. These results align with the histopathological lesions. H3N1 and H6N1 induced faster and more ciliary loss with epithelial cell detachment and damage to the underlying tissue in OOC of both genotypes, especially with H3N1, compared with H9N2-infected BL-OOCs. This supports in vivo studies in adult layers, which showed a nontypical high virulence of H3N1 [5]. Also, the higher viral loads of H6N1 in BL-OOCs go in line with field observations of more severe clinical disease in layers and broiler breeders [6, 34].

Our results partially align with a previous study reporting higher resistance and lower cloacal shedding of H9N2 in infected BL compared with WL [7] suggesting a different density of virus receptors in the magnum across different layer genotypes. Differences were, for example, observed in sialic acid α2.3-gal receptor abundance in the cecum of silkie chickens compared with leghorn white layers [35]. At 24 hpi, both virus strain and host genotype independently impacted viral replication kinetics, and no interaction is suggested. In contrast, a significant virus–genotype interaction was detected at 48 and 72 hpi (*p* < 0.05) (Additional file 3). This suggests that host genotypes may have a different pattern and/or magnitude of regulating virus infection as soon as high viral titers are reached, possibly due to differences in the magnitude and timing of innate and adaptive immunity.

Schön et al. (2021), demonstrated that the unusual virulence of the Belgian H3N1 strain is associated with the NA mutation at position 130 (N130S) [36]. This mutation facilitates recruitment of plasminogen, a protease precursor, enabling HA cleavage and promoting systemic virus spread. Analysis of NA sequences of the investigated AIV strains, as published through the National Center for Biotechnology Information (NCBI) [37] for H3N1, H6N1, and H9N2, revealed corresponding substitutions identified in both H3N1 and H6N1 (N130S, N130H), respectively. These mutations may be attributed to trypsin-independent replication in OOC and use of plasminogen as described previously [36, 38] and can explain the higher viral loads of H3N1 and H6N1, especially in the BL-OOCs compared with H9N2.

IFN λ was previously reported to have a role in mucosal antiviral defense against LPAIVs [12, 39]. However, LPAIVs typically induce a weaker immune response than HPAIVs [43], potentially owing to the differences in the antigenic structure of the *NS1* gene, which is crucial for counteracting the host’s innate immune response, especially interferon production [40]. The NS1 sequence in our strains based on NCBI showed a mutation D171Y in H9N2 which was absent in H3N1 and H6N1 (D171 in these strains) (data not shown). This substitution plays a significant role in reducing the induction of IFN λ and proinflammatory cytokines [41]. Our findings support this as we observed higher IFN λ mRNA expression levels for both H3N1 and H6N1 compared with H9N2 at 48 hpi (*p* < 0.05), correlating with their viral replication kinetics in BL-OOCs [12] We speculate that H3N1 and H6N1 may exhibit resistance to IFN λ enabling higher replication kinetics compared with the H9N2 strain in BL-OOCs. This resistance has been observed with HPAIV H5N1, which employs an evolutionary mechanism through a NS1-mediated IFN signaling block that abrogates the effect of IFN [42]. Differences in the IFN λ response were also observed between genotypes, as BL-OOCs exhibited significantly lower basal levels of IFN λ mRNA compared with WL-OOCs (*p* < 0.05). BL-OOCs showed a remarkably higher fold change increase in IFN λ mRNA expression in response to H3N1 and H6N1 infection compared with WL-OOCs (*p* < 0.05). Our observations suggest that chicken genotypes play an important role in shaping the innate immune response to AIV infection [43]. Virus strain variations were also observed for iNOS expression, H3N1-infected WL- OOCs exhibited higher iNOS levels compared with H9N2-infected OOCs (*p* < 0.05). However, both strains exhibit only a low fold change in iNOS mRNA levels compared with virus-free controls. This finding could explain further the atypical virulence of H3N1 as shown also previously for H5N1 infection, where elevated iNOS expression was associated with tissue inflammation and disease severity [44].

The transcriptomic analysis showed that H9N2 triggered early and higher levels of innate immune-related gene expression than H3N1 at 24 hpi. However, H3N1 induced a higher response by 48 hpi compared with H9N2. The earlier response of H9N2 was characterized by significant upregulation of antiviral genes such as *CCL4*, *IFIT5*, *MX1*, and *LYZ*. These genes were previously linked to resistance against LPAIV H7N1 in chicken lung macrophages derived from resistant compared with susceptible chicken lines following virus infection [31]. These results correlate with the lower viral replication rate of H9N2 compared with H3N1 in the BL-OOCs. Our findings are consistent with a previous in vivo infection study in SPF chickens where H9N2 induced a similar early responses in the spleen which may explain the mild clinical signs observed under field conditions [45]. Interestingly, both strains induced significant upregulation of ISGs such as *IFIT5*, *IFITM3*, *IFITM5*, *OASL*, and *ISG12*-2 at 48 hpi in comparison with virus-free controls while the expression of IFN α, IFN β, and MYD88 was not affected, suggesting the weak role of IFN type I against LPAIV in reproductive tract tissue [12, 46]. The NS1 sequences of both H3N1 and H9N2 strains retain 96E, which is known to preserve NS1 ability to bind and inhibit TRIM25, an interferon activator, and subsequently may explain the very low mRNA expression level of type I interferons (IFN α/β) in our study [47]. For IFN λ, we found discrepancies in the magnitude of expression changes between qRT–PCR and the qPCR array, which can be explained by the fact that qRT–PCR is more sensitive at detecting low-abundance transcripts [48]. At 48 hpi, H9N2-infected OOCs exhibited a significantly higher fold change of *PTX3* and *ISG12-2* compared with H3N1-infected OOCs. *PTX3* is a key early immune responder, previously shown to be strongly upregulated during IBDV infection and after influenza virus H3N2 infection in swine, suggesting a role in early antiviral defense [49, 50]. Similarly, higher expression of *ISG12-2* has been associated with inhibition of Newcastle disease virus replication [51]. The upregulation of both markers in our study may partly explain the lower replication rate of H9N2. This is consistent with the higher expression of *ISG12-2* in lung macrophages of H7N1-resistant chickens, suggesting a role in mediating resistance to influenza virus in chickens. The unusual virulence of H3N1 may be explained by its unique innate immune response profile characterized by significant upregulation of *CCL5*, *PPARG*, *IL6*, and *TLR7* that were not induced with H9N2 infection at 48 hpi compared with virus-free controls. Additionally, H3N1 infection led to higher *CCL4* expression levels as well as lower mRNA *TLR21* expression levels compared with H9N2 at 48 hpi (Figure 5C and D). The impact of virulence on the innate immune response regulation was observed before, when higher mRNA expression levels of *CCL4* and *CCL5* were found in the lungs of chickens infected with highly virulent H5N1 virus, in contrast with lower levels after LPAIV H9N2 infection [48]. *TLR21* contributes to the innate immune response, likely through stimulation of NF-κB activity and the expression of antiviral effectors such as nitric oxide (NO) [17]. Collectively our data suggest various parameters being responsible for higher virulence of H3N1 compared with H9N2.

To our knowledge, this is the first report describing the expression of Imp α3 and α8 in chicken in vitro OOC during LPAIV-host interaction. H9N2-infected BL-OOCs showed lower Imp α3 mRNA levels compared with H3N1-infected and virus-free OOCs, which is correlated with a lower H9N2-replication rate in OOCs of BL chickens, supporting the speculation that Imp α3 expression may be involved in viral pathogenesis. In both genotypes, Imp α8 exhibited a significant downregulation in H3N1- and H6N1-infected OOCs compared with virus-free controls at 72 hpi (*p* < 0.05). We may speculate that the higher virulence of H3N1 in OOCs of BL and WL may help to countermeasure the host defense by inducing cellular shutdown, such as downregulation of imp α8 observed at the later stage of infection [52].

Chemerin mRNA was detected in mice lungs and mesenteric lymph nodes and coincided with increased expression of cytokines such as *IL6* during H1N1 infection [53]. These findings align with our results, where H3N1-infected OOCs showed minor upregulation of chemerin across individual replicates in both genotypes. Further investigations are required to find out if the increase of chemerin mRNA expression in some of the infected OOCs may correlate with a higher viral replication rates and may subsequently associate with reduced reproductive performance of H3N1-infected layers under experimental and field conditions [24].

## Conclusions

Our study highlights the complex interplay between viral strain and chicken genotype in LPAIV reproductive tract infection. In OOCs, the selected H3N1 and H6N1 strains exhibited higher virulence with increased viral loads and more severe endothelial tissue damage compared with H9N2. These parameters were also influenced by the host donor genotype. Higher basal IFN λ expression levels were observed in virus-free WL- compared with BL-OOCs, which may associate with lower replication rates of H6N1 after inoculation of WL- versus BL-OOCs. BL-OOCs mounted faster immune responses to H9N2, whereas H3N1 triggered a delayed but exaggerated innate immune and inflammatory response. The mRNA expression of other host factors such as Imp3, Imp8, and chemerin also varied between chicken genotypes, and can be speculated to contribute to LPAIV control and subsequently disease development. Overall, our results point out the importance of a holistic approach for understanding LPAIV pathogenesis considering both viral- and host-associated factors.

## Supplementary Information

**Additional file 1** **Primers and probes used in the study**. Supplementary table listing the forward and reverse primer sequences used for gene amplification.

**Additional file 2 Genotype-dependent comparison of LPAIVs replication in OOC explants of BL and WL chicken.** Asterisks indicate significant differences in virus replication between genotypes (two-sample t-test). Lowercase letters indicate differences between time points for each virus using ANOVA followed by Tukey HSD all-pairwise comparison test. *p* < 0.05 *n *= 5 / time point for BL. *n *= 8-10 / time point for WL. hpi: hours post-inoculation. H3: H3N1. H9:H9N2. H6: H6N1. BL: brown layer. WL: white layer.

**Additional file 3 Two-way ANOVA of virus strain, genotype and their interaction effects on parameters after LPAIV infection.** All parameters were investigated in OOCs of LB and LSL after infection with LPAIV strains (H3N1, H9N2 and H6N1). IFN: Interferon, iNOS: inducible nitric oxide synthase. *p* < 0.05 was considered statistically significant.

**Additional file 4**  **Differential mRNA expression of IFN λ** **and iNOS in virus-free and LPAIVs-inoculated BL- and WL-OOCs**. IFN λ (A) and iNOS (B) mRNA expression is shown as 40-Ct ± standard deviation (SD). Lowercase letters indicate significant differences between virus-inoculated and virus-free groups at the same time using ANOVA followed by Tukey HSD all-pairwise comparison test. Asterisks indicate significant differences between genotypes (two-sample t-test). *p* < 0.05, *n *= 5 / time point for BL exp, *n *= 5-8 / time point for WL. hpi: hours post-inoculation. H3: H3N1. H9:H9N2. H6: H6N1. NC: virus-free controls. BL: brown layer. WL: white layer.

 **Additional file 5 Differentially expressed genes (DEGs) in response to H3N2 and H9N2 in OOCs at 1, 24, and 48 hpi.** Excel file containing DEGs identified in OOCs infected with H3N2 and H9N2 at different time points (1, 24, and 48 hours post-infection) compared to virus-free controls.

 **Additional file 6 Genotype-associated variation in mRNA expression of importin α3, importin α8 and chemerin.** mRNA expression of importin α3 (A), importin α8 and chemerin (C) was measured in OOC explants of BL and WL after infection with H3N1, H9N2 and H6N1. Lowercase letters indicate significant differences between virus-inoculated and virus-free groups at the same time (two-sample t-test). Asterisks indicate significant differences between genotypes (two-sample t-test). *p*-value is adjusted using Bonferroni correction for multiple comparisons (adjusted α =0.0167). *n* = 5 / time point for BL exp, *n *= 5-8 / time point for WL. hpi: hours post-inoculation. H3: H3N1. H9:H9N2. H6: H6N1. NC: virus-free controls. BL: brown layer. WL: white layer.

## Data Availability

Most data generated or analyzed during this study are included in this published article and its supplementary information files. The datasets not included are available from the corresponding author on reasonable request.

## Abbreviations

*CCL4*: C–C motif chemokine ligand 4
*CCL5*: C–C motif chemokine ligand 5
*CCL20*: C–C motif chemokine ligand 20
*LIPI*: Lipase I
*CXCR4*: C-X-C motif chemokine receptor 4
LYZ: Lysozyme
MX1: Myxovirus resistance 1
*IFIT5*: interferon-induced protein with tetratricopeptide repeats 5
IFITM: interferon-induced transmembrane protein
*IL6*: interleukin 6
*IL15*: interleukin 15
*ISG12-2*: interferon -stimulated gene 12-2
*OSAL*: 2′-5′ oligoadenylate synthase A
*PPARG*: peroxisome proliferator activated receptor gamma
*IL13RA2*: interleukin 13 receptor subunit alpha 2
*NFKB*: nuclear factor kappa B
*NFKBIZ*: *NFKB* inhibitor zeta
*STEAP4*: six transmembrane epithelial antigen of prostate 4
TLR: toll-like receptor
