# Supplementary material for: Virus and host-associated variations in the interaction of low-pathogenic avian influenza viruses with the epithelial target tissue of the chicken reproductive tract
Source: Vet Res. 2026 Jun 25;57:116. doi: 10.1186/s13567-026-01799-7 (PMC13307412; doi:10.1186/s13567-026-01799-7)
Supplement: Supplementary file 1 — Additional file 1 Primers and probes used in the study. Supplementary table listing the forward and reverse primer sequences used for gene amplification. [file 13567_2026_1799_MOESM1_ESM.docx]

**Additional file 1** Primers and probes used in the study

| **RNA Target** | **Primer/Probe (Sequence)** | **GenBank Accession no.** | **Reference** |
| --- | --- | --- | --- |
| Influenza A (M gene) | M-For  AGATGAGTCTTCTAACCGAGGTCG  M-Rev  TGCAAAAACATCTTCAAGTCTCTG  M-probe  (FAM)-TCAGGCCCCCTCAAAGCCGA-(BHQ-1) | MT463331.1 | [28] |
| RPL13 | RPL13 F  GGAGGAGAAGAACTTCAAGGC  RPL13 R  CCAAAGAGACGAGCGTTTG  RPL13 F  (FAM)-CTTTGCCAGCCTGCGCATC-(BHQ-1) | NM_204999.1 | [29] |
| IFN-λ | chIFN λ- For  CATCAGCCCTCTGGGAAAC  chIFN λ-Rev  CTTGGAAGATGTGGAGGATGG  chIFN λ-Probe  (FAM)-ACAGCCAAGAAGAAGGAGACCGC-(BHQ-1) | NM_001128496.1 | [30] |
| iNOS | chINOS-For  CAACAGGAACCTACCATCTGAC  chINOS-Rev  GACCACTGGATTCTCCCAATAC  chINOS-Probe  (FAM)-ACTGATCTTTGCTGCCAAACAGGC-(BHQ-1) | NM_204961 | [30] |
| ch-Importin α3 | ch-Importin α3- For  CCCTCATTTGGTTCCTCTTCTC  ch-Importin α3- Rev  GAACTACCTGTGTCTGCTCATC  ch-Importinα3- Probe  (FAM)-CGCTGAGAGCGGTAGGAAACATTGT-(BHQ-1) | NM_001007963.2 | [21] |
| ch-Importin α8 | ch-Importin α8- For  CATCCAGATCGTGGTGGATAC  ch-Importin α8-Rev  ATCAGTCCCTGTAACCACATTC  ch-Importin α8-Probe  5-(FAM)-TGATTGTCATGACTCCAGCTCTGCG-(BHQ-1)-3 | XM_046901033.1 | This study |
| ch-chemerin | ch-chemerin-For  GGTGCTGGACAAGTACTACAA  ch-chemerin-Rev  CTGGCAGATAGAGGACATCAC  ch-chemerin-probe  3-(FAM)TCGTCACGGTGCTTGATGTCCTTC-(BHQ-1)-5 | NM_001277476.2 | This study |

For: forward primer; Rev: reverse primer. BHQ-1: 6-carboxyfluorescein. TAMRA: carboxytetramethylrhodamine.

Ch: chicken. iNOS: inducible nitric oxide synthase. RPL13:60S ribosomal protein L13. IFN-λ: interferon lambda
