# Supplementary figures and images for: Virus and host-associated variations in the interaction of low-pathogenic avian influenza viruses with the epithelial target tissue of the chicken reproductive tract
Source: Vet Res. 2026 Jun 25;57:116. doi: 10.1186/s13567-026-01799-7 (PMC13307412; doi:10.1186/s13567-026-01799-7)

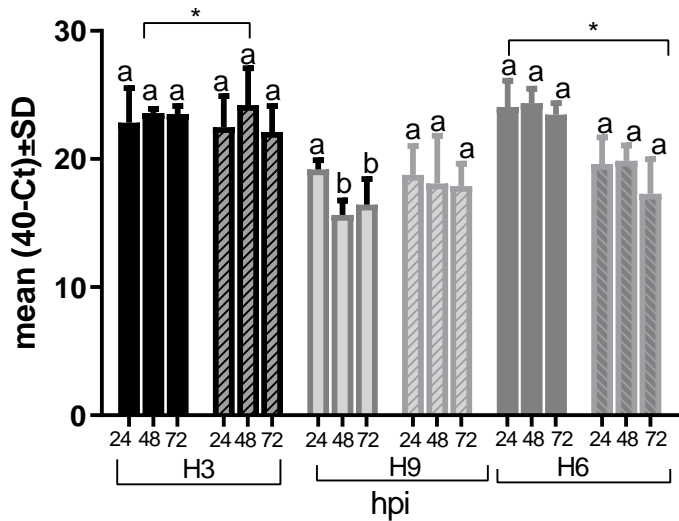

■ H3 BL    ▨ H3 WL    □ H9 BL    ▩ H9 WL    ■ H6 BL    ▨ H6 WL

Supplement: Supplementary file 2 — Additional file 2 Genotype-dependent comparison of LPAIVs replication in OOC explants of BL and WL chicken. Asterisks indicate significant differences in virus replication between genotypes (two-sample t-test). Lowercase letters indicate differences between time points for each virus using ANOVA followed by Tukey HSD all-pairwise comparison test. p < 0.05 n = 5 / time point for BL. n = 8-10 / time point for WL. hpi: hours post-inoculation. H3: H3N1. H9:H9N2. H6: H6N1. BL: brown layer. WL: white layer. [file 13567_2026_1799_MOESM2_ESM.pdf]

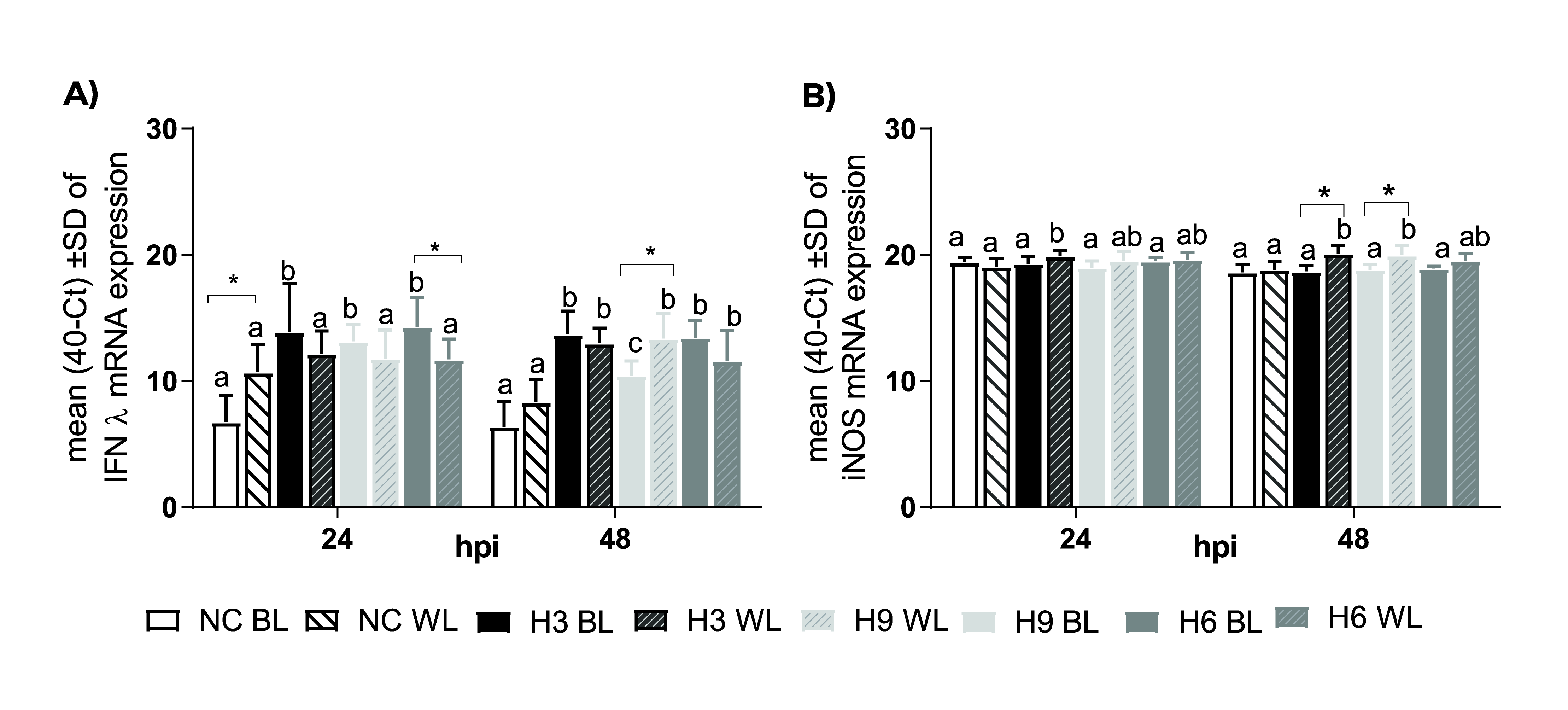

Supplement: Supplementary file 4 — Additional file 4 Differential mRNA expression of IFN λ and iNOS in virus-free and LPAIVs-inoculated BL- and WL-OOCs. IFN λ (A) and iNOS (B) mRNA expression is shown as 40-Ct ± standard deviation (SD). Lowercase letters indicate significant differences between virus-inoculated and virus-free groups at the same time using ANOVA followed by Tukey HSD all-pairwise comparison test. Asterisks indicate significant differences between genotypes (two-sample t-test). p < 0.05, n = 5 / time point for BL exp, n = 5-8 / time point for WL. hpi: hours post-inoculation. H3: H3N1. H9:H9N2. H6: H6N1. NC: virus-free controls. BL: brown layer. WL: white layer. [file 13567_2026_1799_MOESM4_ESM.tif]

A)

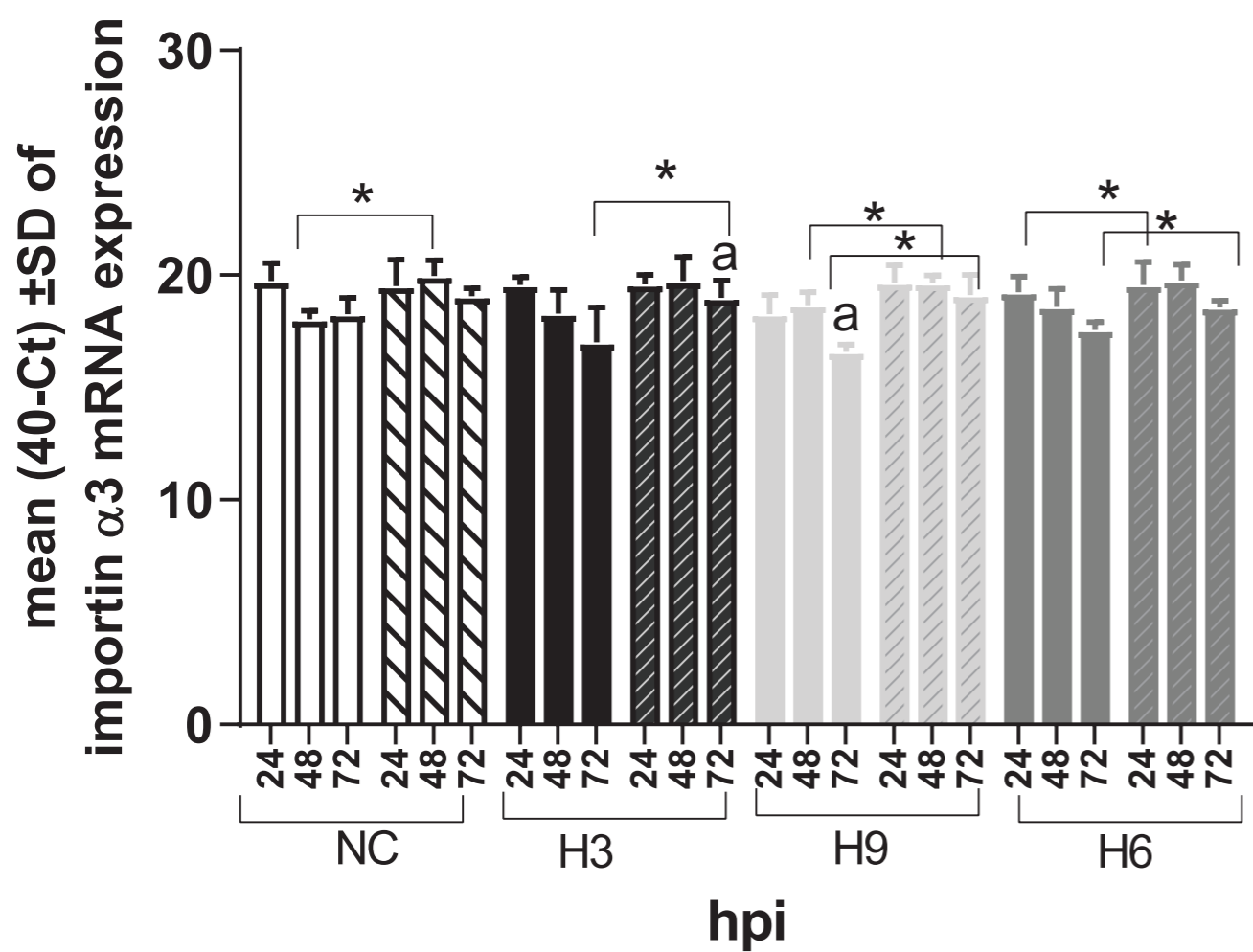

B)

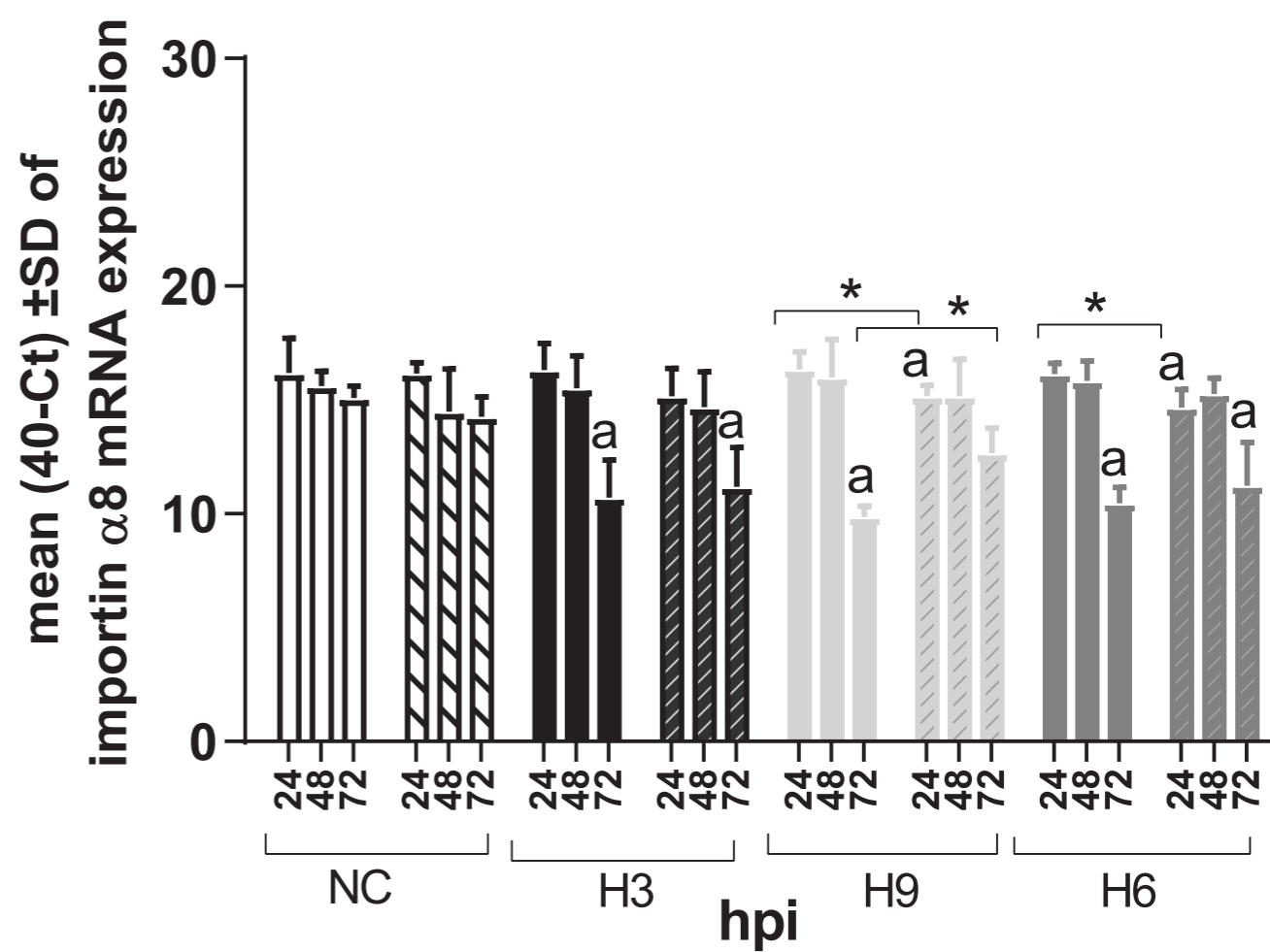

C)

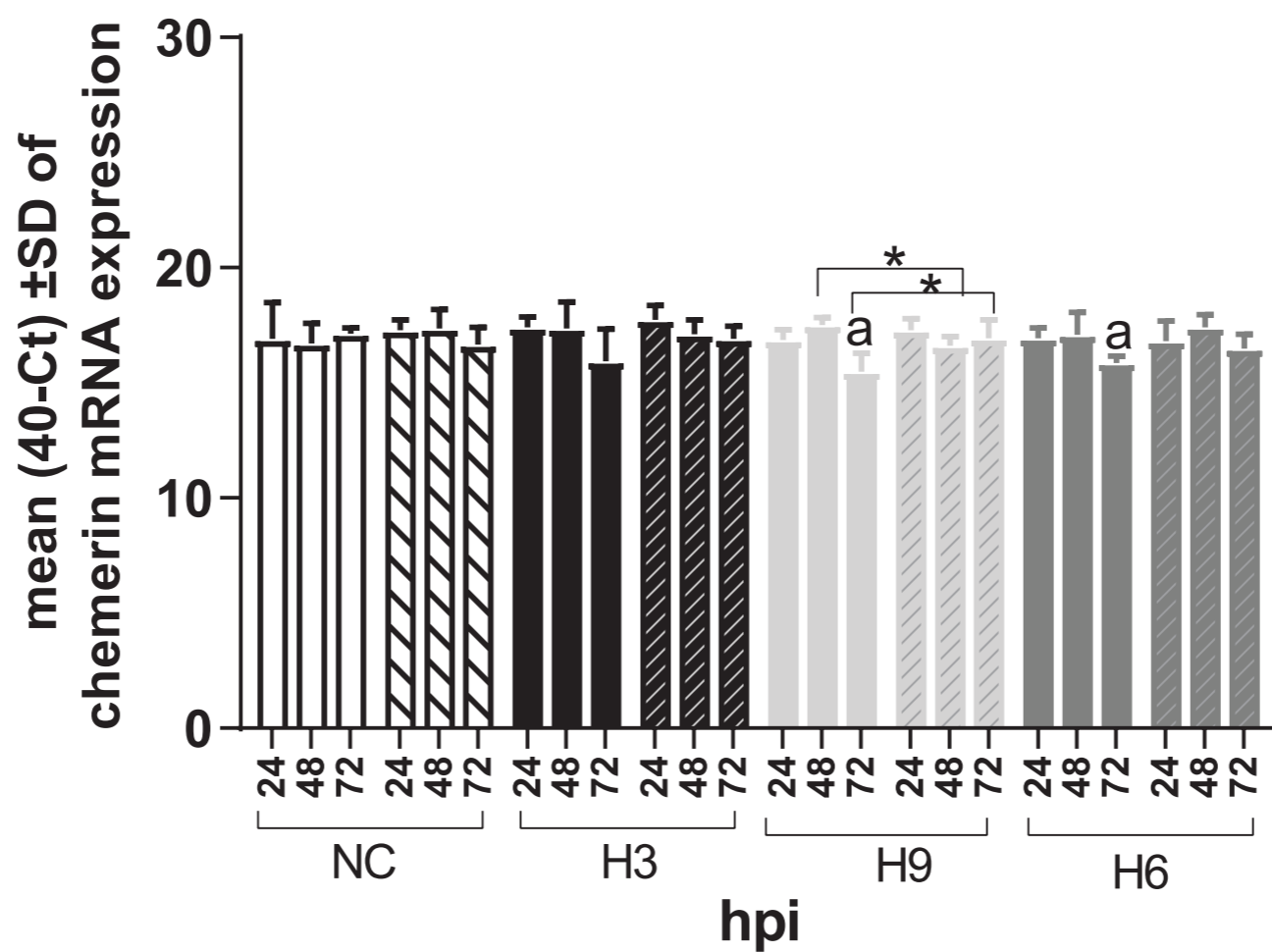

Supplement: Supplementary file 6 — Additional file 6 Genotype-associated variation in mRNA expression of importin α3, importin α8 and chemerin. mRNA expression of importin α3 (A), importin α8 and chemerin (C) was measured in OOC explants of BL and WL after infection with H3N1, H9N2 and H6N1. Lowercase letters indicate significant differences between virus-inoculated and virus-free groups at the same time (two-sample t-test). Asterisks indicate significant differences between genotypes (two-sample t-test). p-value is adjusted using Bonferroni correction for multiple comparisons (adjusted α =0.0167). n = 5 / time point for BL exp, n = 5-8 / time point for WL. hpi: hours post-inoculation. H3: H3N1. H9:H9N2. H6: H6N1. NC: virus-free controls. BL: brown layer. WL: white layer. [file 13567_2026_1799_MOESM6_ESM.pdf]
