# Supplementary material for: Virus and host-associated variations in the interaction of low-pathogenic avian influenza viruses with the epithelial target tissue of the chicken reproductive tract
Source: Vet Res. 2026 Jun 25;57:116. doi: 10.1186/s13567-026-01799-7 (PMC13307412; doi:10.1186/s13567-026-01799-7)
Supplement: Supplementary file 3 — Additional file 3 Two-way ANOVA of virus strain, genotype and their interaction effects on parameters after LPAIV infection. All parameters were investigated in OOCs of LB and LSL after infection with LPAIV strains (H3N1, H9N2 and H6N1). IFN: Interferon, iNOS: inducible nitric oxide synthase. p < 0.05 was considered statistically significant. [file 13567_2026_1799_MOESM3_ESM.docx]

**Additional file 3** Two-way ANOVA of virus strain, genotype and their interaction effects on parameters after LPAIV infection

| Investigated parameters | Time points (hpi) | Virus effect (*p*-value) | Genotype effect (*p*-value) | Virus*genotype interaction (*p*-value) |
| --- | --- | --- | --- | --- |
| Virus replication (Ct-value) | 24 | 0.0019 | 0.0225 | 0.0524 |
|  | 48 | <0.0001 | 0.8592 | 0.0012 |
|  | 72 | <0.0001 | 0.0598 | 0.0135 |
| IFN λ (log2-fold change) | 24 | 0.7701 | <0.0001 | 0.8821 |
|  | 48 | 0.1763 | 0.0061 | 0.0099 |
| iNOS (log2-fold change) | 24 | 0.2117 | <0.0001 | 0.5210 |
|  | 48 | 0.7457 | 0.0018 | 0.3225 |
| Importin α3 (log2-fold change) | 24 | 0.2707 | 0.0107 | 0.1837 |
|  | 48 | 0.9003 | 0.0239 | 0.8043 |
|  | 72 | 0.9167 | 0.0067 | 0.2538 |
| Importin α8 (log2-fold change) | 24 | 0.2768 | 0.0011 | 0.5524 |
|  | 48 | 0.9517 | 0.2290 | 0.8608 |
|  | 72 | 0.7934 | 0.0003 | 0.2384 |
| Chemerin (log2-fold change) | 24 | 0.0333 | 0.4693 | 0.5557 |
|  | 48 | 0.7879 | 0.0042 | 0.1977 |
|  | 72 | 0.7050 | <0.0001 | 0.5362 |

All parameters were investigated in OOCs of LB and LSL after infection with LPAIV strains (H3N1, H9N2 and H6N1). IFN: Interferon, iNOS: inducible nitric oxide synthase. *P* <0.05 was considered statistically significant.
